# U-DCS: characterization of the first permanent human dendritic sarcoma cell line

**DOI:** 10.1038/s41598-020-77471-7

**Published:** 2020-12-04

**Authors:** Kevin Mellert, Julian Benckendorff, Frank Leithäuser, Katarzyna Zimmermann, Peter Wiegand, Giada Frascaroli, Michaela Buck, Muriel Malaise, Gunther Hartmann, Winfried Barchet, Daniel Fürst, Joannis Mytilineos, Regine Mayer-Steinacker, Andreas Viardot, Peter Möller

**Affiliations:** 1 Institute of Pathology, University Hospital of Ulm, Albert-Einstein-Allee 23, 89081 Ulm, Germany; 2 Institute for Forensic Medicine, University Hospital Ulm, Ulm, Germany; 3 Institute of Virology, University Hospital Ulm, Ulm, Germany; 4 Department of Pediatric Hematology, Oncology and Stem Cell Transplantation, University of Regensburg, Regensburg, Germany; 5 Institute for Clinical Chemistry and Pharmacology, University of Bonn, Bonn, Germany; 6 Institute of Clinical Transfusion Medicine and Immunogenetics, German Red Cross Blood Transfusion Service, Baden Württemberg-Hessen, Ulm, Germany; 7 Department of Internal Medicine 3, University Hospital Ulm, Ulm, Germany

**Keywords:** Cancer models, Sarcoma, Cancer

## Abstract

A dendritic cell sarcoma cell line, U-DCS, was established from a dendritic cell sarcoma in a 53-year-old Caucasian male patient. Since its establishment, U-DCS has maintained stable phenotypic characteristics in vitro and has a doubling time of approximately 2 days under standard culture conditions. U-DCS is growing with typical dendritic cell morphology in tissue and expresses the dendritic cell sarcoma immunophenotypic markers S100 protein, MHCI, MHCII, and vimentin. Expression analysis revealed transcripts for the toll-like receptors TLR3, -4, -9 and DDX58 (RIG-I), but not for TLR2. U-DCS shows functional features of dendritic cells with the ability of phagocytosis and antigen-specific T cell stimulation. Karyotype-, CGH-, and mFISH analysis point to a chromosomal instability and a hypotetraploid karyotype with approximately 130 chromosomes. U-DCS is the first immortalized human dendritic cell sarcoma cell line and has some morphological and functional features of dendritic cells without dependency on growth factors.

## Introduction

Dendritic cells (DCs) are a unique class of immune cells originating from hematopoietic stem cells. They act as professional antigen-presenting cells and play a key role in initiating and regulating antigen-specific immune responses^1^. DCs can be classified as conventional DCs type 1 (cDC1), conventional DCs type 2 (cDC2), plasmacytoid DCs (pDC), inflammatory DCs and Langerhans cells^2^. Paracortical T-cell areas harboring DCs in lymph nodes have been referred to as interdigitating dendritic cells (IDC) by histologists^3,4^. Most IDCs have a phenotype compatible with cDC2 lineage^2,5–7^. cDC2 are associated with presenting antigens to the CD4^+^ T-Cells and are capable of Th1 and Th17 induction^8,9^. IDCs are thought to be the likely cell of origin of IDC sarcoma (IDCS). IDCS is a dendritic cell neoplasm of spindle to ovoid cells with phenotypic features similar to those of IDC. IDCS is an extremely rare neoplasm and its etiopathogenesis is unknown. Most studies constitute case reports or small series of up to eight cases^10,11^. A pooled analysis of dendritic cell sarcoma by Saygin et al. in 2013 identified 100 IDCS cases in the English literature^12^. IDCS occur predominantly in the sixth decade^11,12^. Lymph node involvement is common, but about half of the patients have an extranodal disease manifestation^11,12^. IDCS can be associated with lymphoid and myeloid neoplasms and convincing examples of transdifferentiation have been published^13–15^. One case has been shown to harbor somatic BRAF V600E mutation^16^. The clinical course is stage-dependent, but generally aggressive, with about half of all patients dying of the disease. We here report the first permanent human IDCS cell line, U-DCS, derived from an IDCS of a male Caucasian patient. Serial passaging of the cells gave rise to a rapidly proliferating cell line, which was characterized by immunocytochemical, molecular, and cytogenetic techniques and was functionally tested for phagocytosis and antigen presentation.

## Material and methods

### Clinical records

The interdigitating dendritic cell sarcoma cell line, U-DCS, was established from the lung metastasis of an IDCS in a 53-year-old male patient. The patient gave his written informed consent for scientific usage of his tumor cells. The patient initially presented with bilateral axillary lymph node involvement. Therapeutic management included right axillary lymph node dissection and radiation therapy to the left axillary and clavicular lymph node stations. Local recurrence necessitated six cycles of chemotherapy (CHOP) and radiation therapy. Lung metastasis occurred 15 months after initial diagnosis. Resection of the right middle lobe was followed by one cycle of high-dose chemotherapy (ICE), autologous stem cell transplantation and ultimately palliative radiation therapy due to painful bony metastasis. The patient died 26 months after the initial diagnosis.

### Cell culture

All cell culture reagents were purchased from Lonza (Basel, Switzerland), except for fetal calf serum, which was purchased from Seromed/Biochrom (Berlin, Germany). The U-DCS cell line was established and propagated in Iscove/RPMI medium (4:1) supplemented with 10% fetal calf serum, 2 mM l-glutamine, 100 U/ml penicillin, 100 µg/ml streptomycin, and Insulin–Transferrin–Sodium Selenite Supplement (ITS) from Roche (Mannheim, Germany), and maintained at 37 °C in a 5% CO_2_ atmosphere. Subcultures were made with trypsin/EDTA according to standard procedures. Mycoplasma contamination was not detected in any passage. Peripheral lymphocytes of the patient were separated from peripheral blood with Ficoll (GE Healthcare, Munich, Germany) according to standard procedures and cultured in medium with Epstein-Barr virus (produced by the marmoset B-lymphoblastoid cell line B95-8) and 1 µg/ml cyclosporin A (Sandoz, Basel, Switzerland) until B-cell transformation occurred. This cell line was named LCL-U-DCS. All work was performed in concordance with the Declaration of Helsinki and the study was approved by the local ethics committee (Ethics committee of Ulm, vote 369/17). Produced IL-6 and IL-8 was measured in cell free supernatants after 24 h of co-culture of U-DCS cells and T cells by Enzyme-linked immunosorbent assays (ELISA) according to the manufacturer’s handbook (BD Opteia Elisa Sets, BD Bioscience, Heidelberg, Germany; Supplementary Fig. 2).

### Short tandem repeat analysis (STR analysis)

DNA was extracted from the LCL-U-DCS cell line of the patient and from the U-DCS cell line at different passages, using the magnetic bead-based Maxwell-DNA IQ extraction protocol (Promega, Mannheim Germany). DNA extracts were diluted in 50 μl extraction buffer. PCR amplification was carried out with the self-developed Q11-STR kit, and two commercial STR kits, MPX-4 (Serac, Bad Homburg, Germany) and PowerPlex ESX 17 (Promega, Mannheim, Germany) according to the manufacturer’s recommendations. The three STR kits Q11, MPX4, and PowerPlex ESX 17 included the same STR markers: D3S1358, FGA, D8S1179, D18S51, D21S11, TH01, VWA, SE33, D2S1338, D16S539, D19S433, and the gender-typing system Amelogenin. PowerPlex ESX 17 contained the additional STRs D10S1248, D1S1656, D22S1045, D2S441, and D12S391. These kits are commonly used in forensic investigations for human identification and provide a very high probability of identity of 1 in 10^13^ (Q11 and MPX4) and 1 in 10^18^ (PowerPlex ESX 17).

Electrophoresis of the PCR products was carried out on an ABI Prism 3130 Genetic Analyzer (Applied Biosystems, Darmstadt, Germany) with the polymer POP7. The data was analyzed using the GeneMapper ID Software v3.2.

### High resolution HLA-typing

HLA typing was performed with sequence based typing (SBT, DRK-BSD Baden-Wuerttemberg-Hessen, Frankfurt, Germany) for HLA-class I and with sequence specific oligonucleotide probes (SSO, One Lambda Labtype, Canoga Park, CA, USA) for HLA-class II using reagents certified for clinical typing.

### Cytogenetic analyses

To prepare the chromosomes of U-DCS, the cells were incubated for 2 h with 0.15 µg/ml Colcemid (Fluka Biochemika, Neu-Ulm, Germany). Then, the cells were trypsinized, centrifuged and the resulting pellet was resuspended in 1 ml of culture media. The cell suspension was slowly filled up with hypotone solution to a total volume of 10 ml and incubated at 20 °C for 20 min. After pelleting and resuspending the cells in 1 ml of hypotone solution, fixation was performed by slowly adding fixative solution (3 parts methanol + 1 part glacial acetic acid) to a final volume of 10 ml. The cells were pelleted for 5 min at 1000 rpm in a cooling centrifuge at 4 °C. The pellet was resuspended in 1 ml fixation solution. Metaphase preparation and G-band staining was performed using standard protocols.

### Comparative genome hybridization (CGH)

Chromosomal gains or losses were analyzed using standard CGH techniques. DNA derived from U-DCS cells was Nick-translated and labelled with a red fluorescing dye (Cy5). Then, control metaphase chromosomes were co-incubated with the red fluorescing cell line DNA and green fluorescing control DNA (Abbott, Wiesbaden, Germany) and analyzed using an Axioplan 2 microscope (Zeiss; Jena, Germany). Gains and losses were identified by comparing the green and red fluorescence signals (ISIS3; MetaSystems; Altlussheim, Germany).

### Multicolor FISH

For multicolor FISH (mFISH) analyses, previously prepared metaphase chromosomes of U-DCS were stained using the 24xCyte Multicolour Probe Kit (Metasystems, Altlussheim, Germany). The fluorescence chromosome painting was performed according to the manufacturers’ handbook and examined using a specialized filter system as recommended to detect the different fluorescence combinations. In short, the metaphase chromosome slides were incubated for 30 min in twofold saline sodium citrate (SSC) buffer (70 °C), cooled down for 20 min and denatured in 0.07 N NaOH for 1 min. After a further incubation in 0.1- and 2-fold SSC (4 °C) the slides were incubated in rising percentages of ethanol (70%, 95%, 100%, 1 min each) and air dried. Subsequently, the chromosomes were incubated for 2 days with the denatured probe cocktail in a humidified chamber at 37 °C. After that, the slides were washed and counterstained with 4′,6-Diamidin-2-phenylindol (DAPI; 1 µg/ml) and finally analyzed using a fluorescence microscope (Axioplan 2, Zeiss, Germany).

### Immunophenotyping

Primary antibodies used for staining of frozen and paraffin sections from the tumor tissue and the resulting cell lines are summarized in Table 1. Detection of the primary antibodies was performed using rabbit anti-mouse or goat anti-rabbit IgG peroxidase-conjugated secondary antibodies in combination with the RED detection reagents according to the manufacturers’ handbook (RED detection kit, Dako, Copenhagen, Denmark). The staining was evaluated by a pathologist while simultaneously comparing it to appropriate positive and negative controls. The intensity of the staining was graded as strongly positive (++), positive (+), weakly positive (+/−), or negative (−).

**Table 1:** Expression pattern of immunophenotypic markers in the patient’s IDCS (parental tumor) and the respective cell line U-DCS. The used antibodies and specific clones are given. The results are illustrated as strongly positive (++), positive (+), weakly positive (+/−), negative (−). *n.d. *not determined due to limited amounts of tumor tissue.

Antibody	Clone	Parental tumor	U-DCS
CD1a	O10	−	−
CD1c	2F4	n.d.	−
CD11c	5D11	+/−	−
Clec10a	OTI2B10	n.d.	−
CD14	ERP36532	n.d.	−
CD21	1F8	−	−
CD23	SP23	−	−
CD25	ILR.1	+	+
CD35	Ber-Mac-drc	−	−
CD40	(Polyclonal)	−	−
CD54	84H10	+	+
CD68	KP1	+	+
CD80	BB1	+	+
CD83	HB15e	+	+
CD86	IT2.2	+/−	−
CD123	BR4MS	−	−
CD205	Dec205	+/−	+/−
CD207	12D6	−	−
Factor VIII	(Polyclonal)	−	−
Lysozyme	(Polyclonal)	−	−
MHCI	W6/32	++	++
MHCII (HLA-DR)	1B5	++	++
Podoplanin	D2-40	−	−
S100	(Polyclonal)	+	++
Vimentin	Vim3B4	++	++

### Tests for endogenous viruses

We tested the cell line for the most common endogenous viruses. Test methods and primer sequences for the PCR assays used are listed in Supplementary Table 1.

### Maturation of U-DCS cells

To induce differentiation of U-DCS cells and to test whether they mature like dendritic cells, cells were seeded in 25 cm^2^ culture flasks (Nunc, ThermoFisher Scientific; ~ 50% confluency) and allowed to adhere overnight. Following the cells were incubated with media containing a dendritic cell maturation cocktail [TNFα: 10 ng/ml (ImmunoTools, Friesoythe, Germany), IL-1β: 10 ng/ml (ImmunoTools)^17^, IL-6: 15 ng/ml (ImmunoTools), and PGE2: 1 µg/ml (Santa Cruz Biotechnology, Dallas, USA)]. After 24 h, cells were detached and cells blocks were made. Sections of these blocks were immunostained for CD1a, CD1c, CD11c, CD14, CD68, CD207, HLA-ABC, HLA-DR and Clec10a.

### Phagocytosis assay

Cells were grown in 1 cm^2^ wells with 500 µl culture medium for 24 h, washed once with medium and then incubated with 50 µl FITC-latex beads (0.1 µm particle size, Cayman Chemical Company, Ann Arbor USA) for 12 h. Alternatively, cells were incubated for 6 h with isolated PBLs (small cell suspension fraction of previously cryo-preserved peripheral blood mononuclear cells (PBMC) isolated from buffy-coats, according to standard Ficoll density gradient protocols). After incubation, cells were washed two times in PBS, detached with trypsin/EDTA, centrifuged at 600 rpm for 3 min and deposited onto glass slides using a cytocentrifuge (Shandon, Pittsburgh, PA, USA). Then the slides were fixed in 100% ethanol for 1 min and air-dried. PBL incubated cells were counterstained with Hematoxylin. Latex bead incubated cells were stained with anti-HLA-DR and the secondary antibody goat anti mouse-Cy3 (Jackson Immunoresearch, Newmarket, UK) according to standard immunocytochemical protocols, counterstained with DAPI (1 µg/ml) and mounted with Vectashield (Vector Laboratories, Burlingame, USA). The slides were analyzed using an Axioscope-2 microscope (Zeiss, Germany) equipped with appropriate filter sets (AHF, Tübingen, Germany), the CCD camera Jai-M 300 and the ISIS software from Metasystems (Altlussheim, Germany).

### Mixed leukocyte reaction assay (MLR)

MLR was performed according to Ratta et al.^18^ and was partly described earlier^19^. In short, U-DCS cells were left untreated, incubated with a multiplicity of infection (MOI) of 5 of human cytomegalovirus (HCMV) or stimulated with 100 µg/ml Tetanus toxoid (Statens Serum Institut, Copenhagen, Denmark). Cells were collected 1 day later, extensively washed, irradiated (50 Gy) and plated in decreasing numbers with previously cryo-preserved allogenic peripheral blood mononuclear cells (PBMC) isolated from three HCMV-seronegative and three HCMV-seropositive (tested by Vidas CMV IgG, Biomerieux, France) buffy-coats, according to standard Ficoll density gradient protocols. PBMC were thawed, washed, resuspended in RPMI containing 5% human AB serum (Institut für Klinische Transfusionsmedizin und Immungenetik Ulm GmbH) and plated in triplicate at 1 × 10^5^ cells per well in a 96-well-U-bottom plate (Corning, NY). Increasing ratios of stimulators U-DCS and responder PBMC (stimulators: responders from 1:513 to 1:8) were co-cultured for 5 days and subsequently pulsed with 1 μCi/well [^3^H] thymidine for 18 h. Proliferation was determined by measuring the [^3^H] thymidine incorporation in a β-counter (Wallac MicroBeta TriLux, Perkin Elmer, Rodgau, Germany) and calculating the stimulation index (SI) as follows: SI = counts per minute (cpm) (PBMC + stimulators) / cpm (PBMC alone).

### Measurement of the expression of TLR2, -3, -4, -9 and DDX58

To measure (end-point) the expression of TLR2, -3, -4, -9, and DDX58 (RIG-I) in U-DCS cells, total RNA was extracted using the RNeasy RNA purification kit (Qiagen, Hilden) as described in the manufacturers’ handbook. RNA was reverse transcribed into cDNA (Superscript III; Invitrogen, Karlsruhe, Germany). Target sequences were amplified using a touchdown PCR program (Initial denaturation 5 min 95 °C, followed by two cycles 95 °C 30 s. 62 °C 30 s, 70 °C 30 s. The next two cycles were 95 °C 30 s, 60 °C 30 s, 70 °C 30 s. The next two cycles were 95 °C 30 s, 58 °C 30 s, 70 °C 30 s. Main 30 cycles were carried out at 95 °C 1 min, 56 °C 30 s, 70 °C 30 s, and finally 70 °C for 10 min). Primer sequences are given in Supplementary Table 2.

### Statistics

To test for statistical significant differences between test groups 2-way ANOVA was performed (Prism 6, Graphpad Software Inc, SanDiego, CA, USA). p values < 0.05 was considered significant (*p < 0.05; **p < 0.01; ***p < 0.001).

## Results

### U-DCS shows a morphology suggestive of conventional dendritic cells

**Figure 1:**
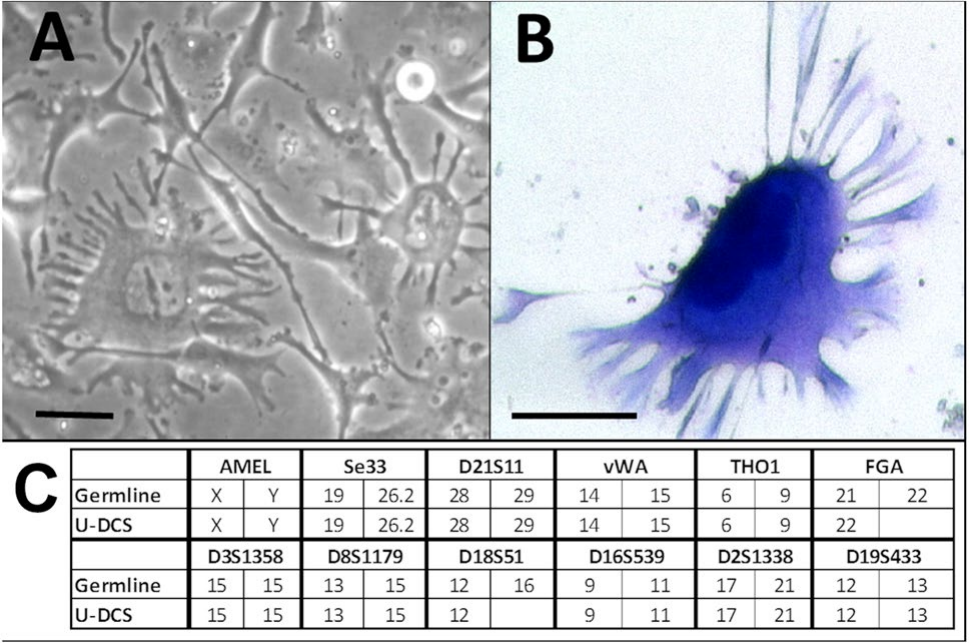
Morphology and short tandem repeat analysis of the cell line U-DCS. (**A**) Phase contrast picture of living U-DCS cells in vitro. (**B**) May–Grünwald Giemsa staining of a single U-DCS cell. The black bar indicates a length of 40 µm. (**C**) Short tandem repeat analyses. The origin of the established cell line (U-DCS) was confirmed by comparing the detected alleles of 12 markers. The allele numbers are given.

The U-DCS cell line grows adherent to standard culture flasks and has a cytomorphology suggestive of conventional dendritic cells in tissue. Most prominent they show lots of branched cytoplasmic projections as typically seen in cDCs (Fig. 1A,B). The cells are extremely large: a single globose cell in mitosis has a diameter of 50 µm and an interphase cell is approximately 200–400 µm in size. They seem to have no lateral contact and detach easily with trypsin/EDTA in a single-cell suspension. Population doubling time is approximately 2 days, strongly dependent on the cells’ confluence. U-DCS has maintained the same stable phenotypical morphology for more than 50 population doublings in vitro.

### Short tandem repeat analyses confirm that the cell line originates from the patients’ tumor

A major challenge for cell culture is the fact that intraspecies cross-contaminants or even interspecies contaminants of cell lines occur at a high rates^20–22^. To demonstrate the genetic identity of the derived cell lines we characterized U-DCS by DNA profiling with polymorphic short tandem repeat (STR) markers to validate the typing results. The results of the STR analyses indicate in U-DCS a loss of heterozygosity in FGA and D18S51, compared with the autogenous lymphoblastoid cell line (“germline”; Fig. 1C). No further STR alteration occurred after 2 years in culture.

### HLA typing

High resolution HLA typing showed HLA-A*11:01,*24:02, HLA-B*15:01,*35:01 and HLA-C*03:03,*04:01 for HLA-class I as well as HLA-DRB1*01:01,*13:02 and HLA-DQB1*05:01,*06:04 for HLA-class II. This HLA-phenotype is fairly common in Caucasian populations with an expected frequency of about 2 per million based on published haplotype frequencies^23^.

### U-DCS cells express typical dendritic cell sarcoma immunophenotypic markers

In the frozen sections, the patient’s IDCS were identified as MHCI- and MHCII-positive cells, co-expressing CD68, CD80 and showing partial expression of CD11c, CD86, CD205 and S100 protein (Table 1). The derived cell line U-DCS has a similar expression pattern apart from the fact that U-DCS cells show complete strong expression of S100 protein, a complete minor expression of CD205 and loss of CD11c and CD86. Further immunophenotypic characterization of U-DCS cells revealed expression of the markers CD25 (cytoplasmic), CD54, and CD83. Immunophenotypic markers not expressed were CD1a, CD 1c, CD11c, CD14, CD21, CD23, CD86, CD123, CD207, Factor VII, Lysozyme, Clec10a, and Podoplanin (for pictures of some key stainings see Supplementary Fig. 1).

**Figure 2:**
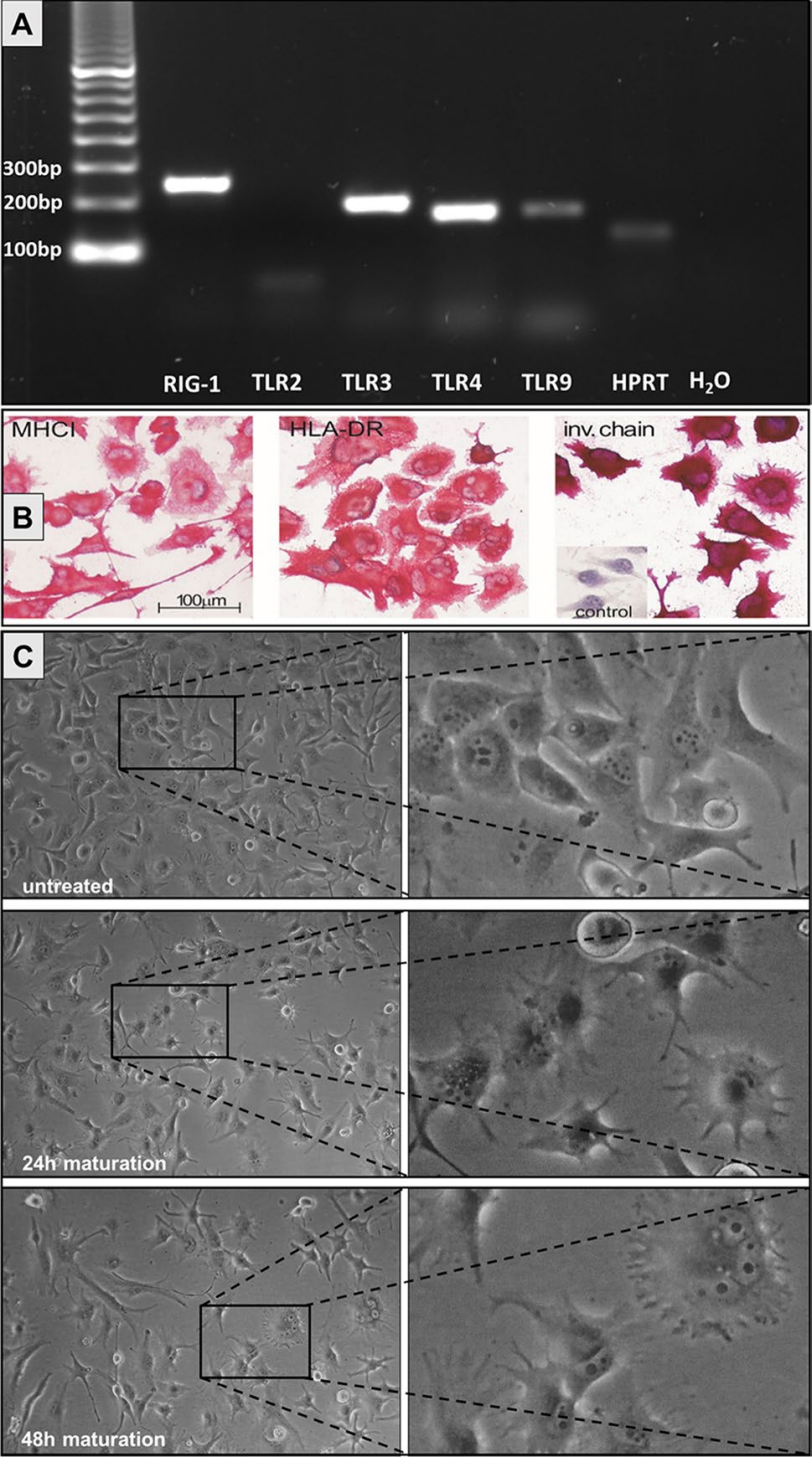
Expression of PRRs and MHCs in U-DCS cells and phenotypical analyses. (**A**) Agarose gel electrophoresis of PCR products of primers targeting transcripts of RIG-1, TRL2, -3. -4, -9 and HPRT. No expression of TLR2 was detected. (**B**) Immunocytochemistry of U-DCS cells to detect MHCI, HLA-DR and the invariant chain (cytoplasmatic). The cut-out (“control”) shows the result of a control experiment without primary antibodies. (**C**) Maturation of U-DCS cells. Cells were either incubated in standard cell culture media (upper panel) or incubated with media containing the dendritic cell maturation reagents (TNFα: 10 ng/ml, IL-1β: 10 ng/ml, IL-6: 15 ng/ml, and PGE2: 1 µg/ml) for 24 h (middle panel) or 48 h (lower panel). Cells show a more dendriform morphology with enhanced numbers of cytoplasmic protrusions after incubation with the maturation cocktail for at least 24 h.

The expression of genes encoding for typical immune-related receptors was tested by endpoint PCR analyses of the reverse transcribed mRNA of U-DCS. It could be shown that U-DCS express TLR3, TLR4, TLR9 and DDX57 (RIG-1) indicating the cells’ ability to recognize pathogen-associated molecular patterns (Fig. 2A). U-DCS cells constitutively secrete the proinflammatory cytokines interleukin 6 (IL-6, about 80 pg/ml) and interleukin 8 (IL-8, about 20,000 pg/ml). Both cytokines are further up-regulated by peripheral T cells (up to 400 pg/ml and 40,000 pg/ml respectively, Supplementary Fig. 2). Immunocytochemical staining revealed strong cytoplasmic and to a varying degree membranous expression of MHC-I and MHC-II in U-DCS (Fig. 2B).

### U-DCS shows evidence of maturation capability

After incubation with maturation reagents for 24 h, cell morphology altered to a more dendritic appearance (Fig. 2C). Furthermore, cells rounded up and could be detached easily from the surface after 30 s incubation with trypsin/EDTA whereas untreated cells needed about 2 min incubation. A small immunochemistry panel staining of synchronized cells with or without maturation cocktail incubation did not reveal significant changes in protein levels. With or without maturation, cells showed a strong positivity of HLA-ABC and HLA-DR, a weak positivity of CD68 and were negative for CD1a, CD1c, CD11c, CD14, CD207, and Clec10a.

### U-DCS was tested negative for multiple endogenous viruses

At admission to the hospital the patient had been tested routinely for the viruses HIV, HBV, HHV-6, HCV, Adenoviruses, Astrovirus, Rotavirus, Norovirus, and Parvovirus B19 (PVB-19) and was proved to be negative for all of them. U-DCS secretes large amounts of IL-8 (Supplementary Fig. 2). IL-8 is a proinflammatory chemokine found in numerous cell types, including monocytes/macrophages and DCs. In cancer it potentiates proliferation of cancer cells, metastases and angiogenesis^24,25^. Since it is known that different viruses can infect DCs and induce elevated IL-8 secretion, we additionally tested the cell line U-DCS for the viruses ADV, CMV, EBV, HHV-6, HPV, and PVB-19. U-DCS was tested to be negative for all of the viruses. Supplementary Table 1 displays the list of viral pathogens tested either in the patient or in the cell line, the test method and the respective primers used in PCR.

### Genetic features of U-DCS

**Figure 3:**
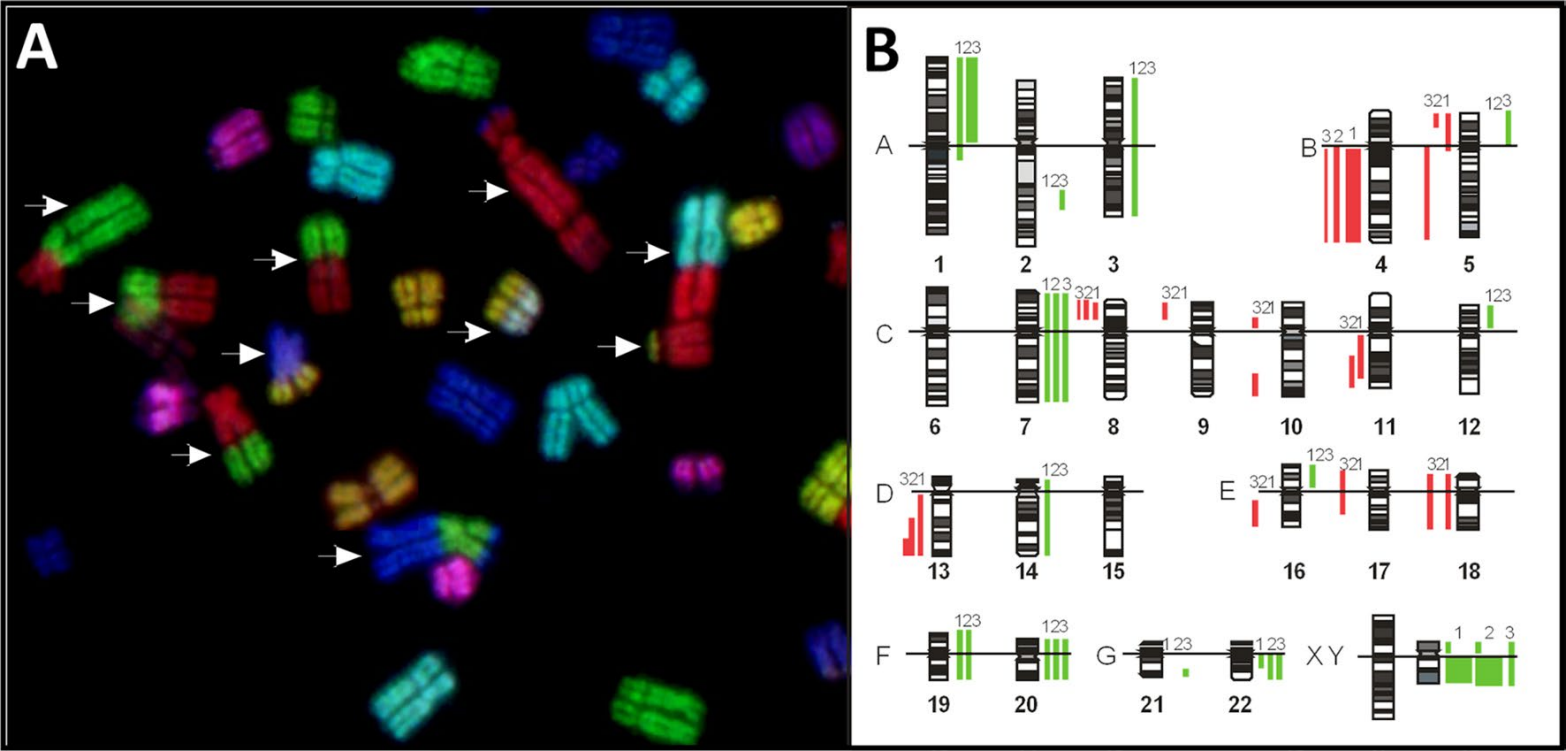
Genetic features of the U-DCS cell line. (**A**) Complex rearrangements in chromosomal structure of U-DCS exemplarily shown in a part of an endoreduplicated metaphase stained using the multicolor FISH technique. Various rearrangements are indicated by white arrows. (**B**) Gains (green) and losses (red) of chromosomal material shown in the original U-DCS (1) and the established lines established independently from 2 different pleural effusions (2, 3).

Chromosomal studies of U-DCS were first performed after 4 months and subsequently at different passages. Karyotype analysis of U-DCS revealed a more than tetraploid karyotype with approximately 130 chromosomes and multiple aberrations. Metaphases stained using the trypsin*-*Giemsa banding (G-banding) technique revealed a highly complex karyotype, in which almost every chromosome appeared modified (not shown). Approximately 5% of the cells were endoreplicated. To demonstrate the complexity of this karyotype, 24-color FISH was performed. Using mFISH techniques, we detected variations in individual chromosome copy numbers, as well as multiple complex chromosomal rearrangements. Figure 3A illustrates a part of an endoreplicated mFISH-stained metaphase. Comparative genome hybridization (CGH) was performed for the cell line U-DCS and two cell lines originating from different pleura effusions (named U-DCSII and U-DCSIII), on 10 metaphases in each case. It revealed the following similarities: ish cgh enh(1p), dim(4q), enh(7), dim(8)(p12 pter), dim(13)(qter q31), enh(20), enh(22)(cen q12), enh(Y) (Fig. 3B).

### U-DCS cells are capable of phagocyting particles and of processing and presenting typical human antigens to leucocytes

**Figure 4:**
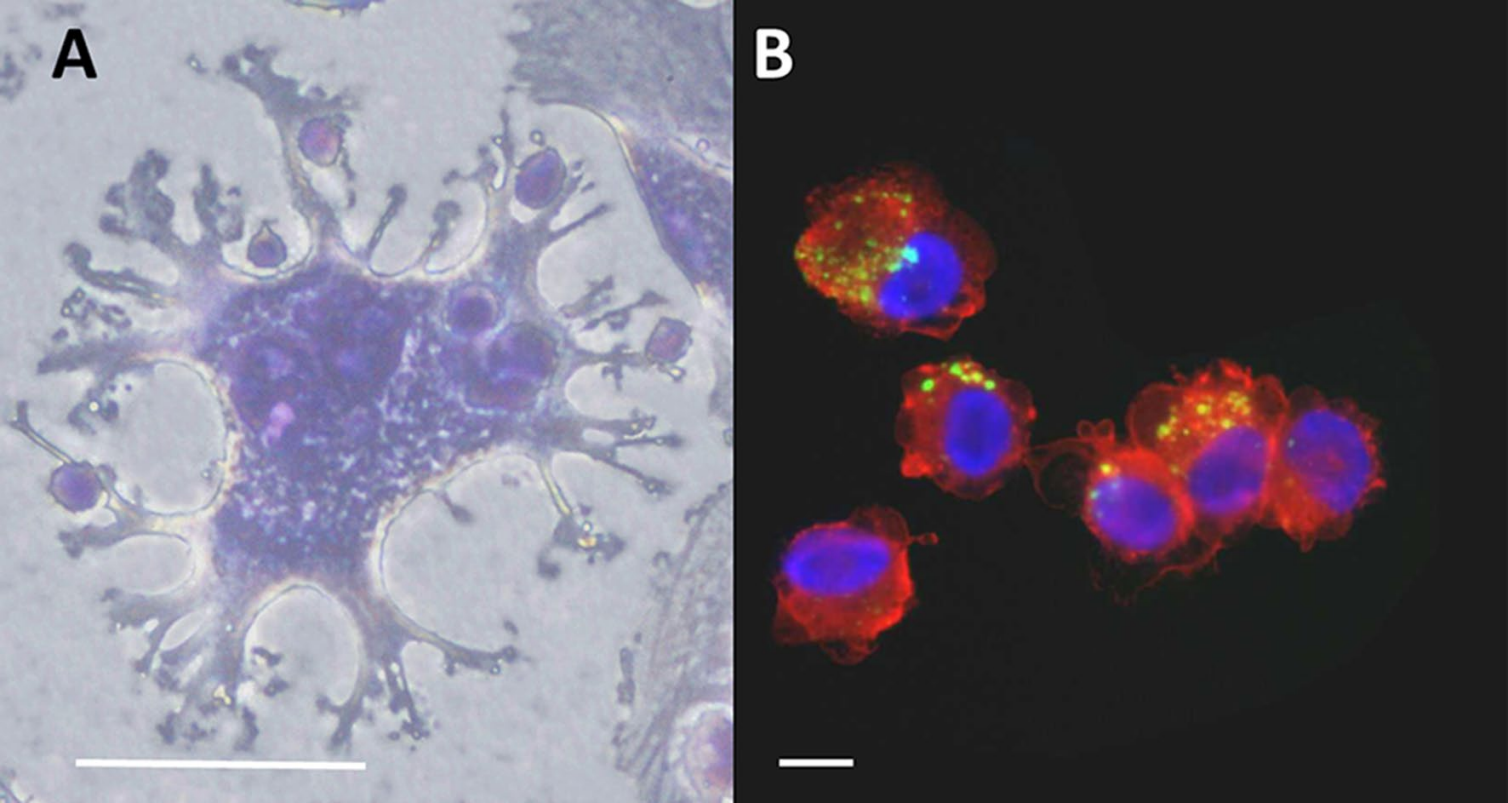
Uptake of PBL and fluorescent latex beads into U-DCS cells. White bars indicate 40 µM of length. (**A**) Phase contrast picture of a U-DCS cell after incubation for 6 h with PBL. The PBLs are taken up into big vesicles. (**B**) Fluorescence image of U-DCS after a 12 h incubation with FITC-tagged latex beads (green). The cells were counterstained with DAPI (blue) and Cy3-labelled anti-goat antibodies targeting the primary HLA-DR antibodies (red).

**Figure 5:**
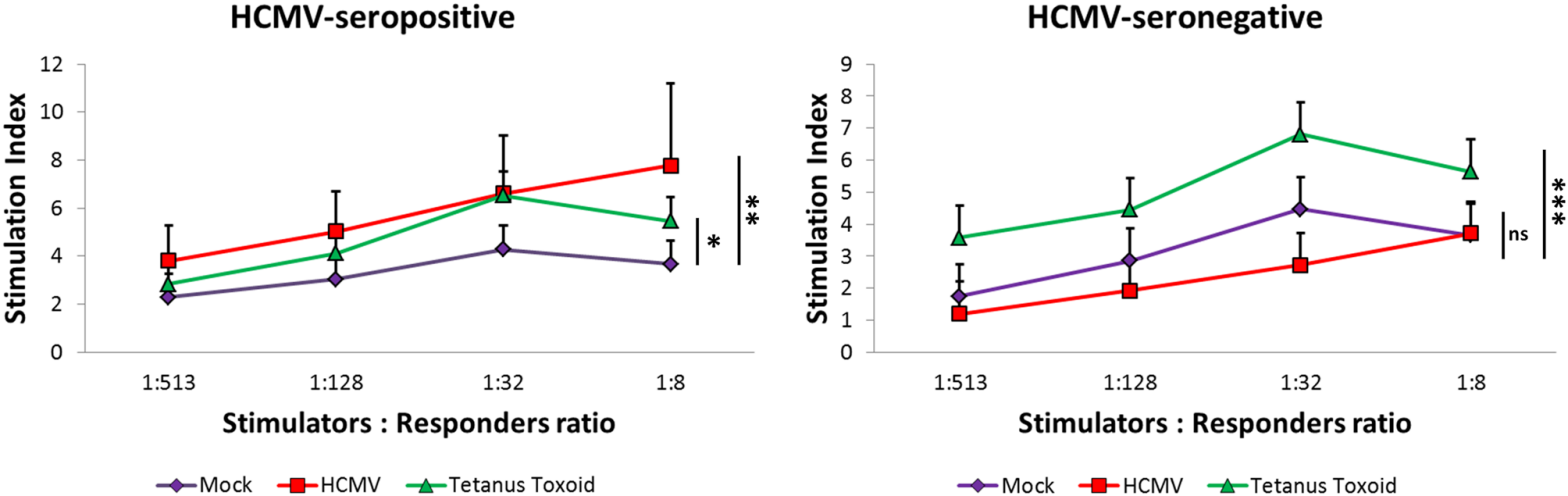
MLR-stimulatory capacity of irradiated U-DCS (see materials and methods for details and stimulation index calculations). Red line, HCMV (human cytomegalovirus) treated U-DCS; green line, TT (Tetanus toxin) treated U-DCS; purple line (mock), untreated U-DCS. The results are presented as the mean + SD of three independent experiments. *ns* no significant differences *p < 0.05, **p < 0.01, ***p < 0.001.

Incubation of the U-DCS cells with fluorescently labeled latex beads for 12 h led to cellular uptake and internalization of the latex beads in cytoplasmic vesicles. U-DCS cells phagocytose latex beads and engulf peripheral blood leucocytes (PBL) fast and efficiently. Figure 4A shows PBL attached to and internalized into U-DCS after 6 h incubation. In Fig. 4B, a cytospin preparation shows U-DCS cells with phagocytosed FITC-labeled latex beads. The cells were counterstained with HLA-DR-Cy3 and DAPI to highlight a cellular membrane marker and the nucleus, respectively. The localization of labeled beads indicates a cellular uptake. We therefore assume that U-DCS cells are able to phagocytose foreign structures. Furthermore, we evaluated whether U-DCS were capable of presenting antigens to T cells. We pulsed U-DCS with common recall antigens and measured the resulting proliferation of T cells in a Mixed Leukocyte Reactions (MLR) assay. Since monocultures of irradiated U-DCS had comparable levels of [^3^H] thymidine incorporation as peripheral blood mononuclear cells (PBMC) (3361 ± 1218 cpm vs. 4852 ± 2367 cmp, respectively) we concluded that irradiation with 50 Gy was sufficient to prevent U-DCS proliferation. As shown in Fig. 5, untreated U-DCS promoted only a small T cell proliferation likely due to the imperfect HLA match. While the recall antigen Tetanus toxoid seemed to be efficiently presented to T cells obtained from all six blood donors (immunization confirmed by Elisa measurement of IgG antibodies against Tetanus). U-DCS pulsed with human cytomegalovirus (HCMV) promoted T cell proliferation only in HCMV-seropositive but not HCMV-seronegative donors, most likely due to an antigen-specific stimulation.

## Discussion

Our results demonstrate that we successfully established a dendritic cell sarcoma cell line, U-DCS, and that U-DCS cells bear morphological and functional resemblance to IDCS and to some extent to conventional dendritic cells. U-DCS is the first human permanent dendritic cell sarcoma cell line derived from an IDCS. We established U-DCS from a lung metastasis and a lymphoblastoid cell line by EBV transformation of peripheral B cells of the patient. By STR analysis we verified the derivation of these cell lines and demonstrated the molecular stability of the tumor cells in vivo and in vitro.

Owing to the fact that IDCS is an extremely rare tumor entity there is no consensus on a standard treatment strategy^11,12^. As in our case, patients are often treated by surgical resection with subsequent chemotherapy or radiation therapy, but the outcome is often poor^11,12,26,27^. Much of our current knowledge on IDCS has been based on a very limited number of scientific studies and case reports. The etiopathogenesis of IDCS is unknown. A viral etiology, particularly infection with EBV and HHV-8 has been excluded^28^. Noteworthy is the association of IDCS with other hematological malignancies like chronic lymphocytic leukemia/small lymphocytic lymphoma^15^ or follicular lymphoma^13^, most likely constituting examples of transdifferentiation.

The reasons for the distinct IL-8 secretion in U-DCS remain unclear. In dendritic cells IL-8 secretion seems to be associated with DC activation and recruitment of pro-inflammatory mediators, particularly neutrophils^29^. IL-8 expression is stimulated by various cytokines (Interleukin-1, Interleukin-6, CXCL12, and TNFα), hypoxia, reactive oxygen species (ROS), bacterial particles and other environmental stresses^30–32^. We tested for multiple endogenous viruses to rule out virus-induced IL-8 secretion. Furthermore, there was no evidence for bacterial contaminants to induce IL-8 secretion. IL-8 is overexpressed in various cancer cell lines^30^. Parallel genome-scale loss of function screens in 216 cancer cell lines implicate that IL-8, CXCR1 or CXCR2 knockdown has a negative impact on cell survival and proliferation^30,33^.

In the present study, we introduce U-DCS as a new model cell line for human IDCS cells. IDCS consistently express the immunophenotypic markers S100 and vimentin, with markers of follicular dendritic cells (CD21, CD23), Langerhans cells (CD1a, CD207), pDC (CD123) and macrophages (CD163) being negative. IDCS are positive for MHC class II (HLA-DR) and weakly positive for CD68, lysozyme and CD45^34^. We demonstrate that both U-DCS and its parental IDCS share these immunohistochemical features (Table 1). Furthermore, the expression of the following markers reinforces the dendritic cell immunophenotype in U-DCS: the adhesion molecule CD54 (ICAM1), which plays a critical role in priming naive T cells^35^, the co-stimulatory molecule CD80, which is upregulated upon DC maturation^5,36^ and constitutes a part of the immunological synapse to activate T cells^37^ and the activation marker CD83, which also seems to be involved in the regulation of DC-mediated T-cell proliferation^38^. U-DCS shows no expression of the costimulatory molecule CD86, which is assumed to be required for T-cell activation^39–42^. The expression of the costimulatory molecule CD25, which may be involved in T cell suppression^43^ was found to be restricted to the cytoplasm^40^.

Immunocytochemical staining demonstrated that HLA-DR is strongly expressed in the cytoplasm of U-DCS. The major function of MHC class II on antigen presenting cells (APCs) is the presentation of peptides derived from extracellular proteins to CD4^+^ T lymphocytes. Its associated invariant chain CD74 is required for the formation, intracellular transport, and internalization of HLA-DR molecules from the cell surface. CD74 is expressed at lower density than HLA-DR on the surface of APC and this low surface expression might be correlated with DC motility^44,45^.

RT-PCR analysis showed that U-DCS cells express transcripts of the pathogen recognition receptors (PRRs) TLR3, -4 -9 and RIG-I, but not TLR2. TLRs and RLRs are PRRs that, upon activation, induce pathways involved in antigen presentation by APCs. Though human DC subsets exhibit common and discriminative PRRs, we couldn’t assign U-DCS cells to a specific DC subset^46–49^. Divergent expression pattern might be due to the neoplastic nature of the U-DCS cells or due to a lack of extracellular stimuli^50^.

IDCS have immunophenotypic characteristics similar to normal IDCs^26^ and show a phenotype compatible with cDC2 lineage^2,5–7^. U-DCS has preserved some central functional features of cDCs: We demonstrated phagocytic ability by incubating the U-DCS cells with fluorescently labeled latex beads. Incubation with PBL led to an attachment and internalization of lymphocytes. MLR assays with U-DCS cells treated with Tetanus toxin and CMV demonstrated antigen specific T cell proliferation.

Furthermore, U-DCS cells clearly displayed morphologic changes of maturation after incubation with maturation reagents and seemed to lose parts of their ability to adhere. Both observations are consistent with cellular DC maturation. Nevertheless, further experiments have to be performed to confirm the cells’ ability to mature.

U-DCS do not express the DC markers CD1a, CD1c, and CD11c but fit well to the immunohistochemistry expression patterns described in iDC sarcomas^51–53^.

U-DCS vaguely resembles the cell line MUTZ-3, first described by Hu et al.^54^ and further characterized by Santegoets et al.^55^ as a model system for differentiation into interstitial DC and Langerhans cells with the help of cytokines. Acute myeloid leukemia cell lines like MUTZ-3, THP-1 or HL-60 might be differentiated to DC-like cells but these lines are likely to retain AML features that may mask important dendritic cell sarcoma features. As U-DCS per se is a model for dendritic cell sarcoma these issues are eliminated. Further studies will be needed to determine whether U-DCS can be assigned to one of the established DC subsets. Due to its origin from a malignant tumor, U-DCS is likely to display some deviation from non-neoplastic DCs, and accordingly might be difficult to classify. Although concessions are surely necessary, we are confident that this cell line could foster IDCS research.

## Supplementary information

Supplementary Information.
