# Supplementary material for: U-DCS: characterization of the first permanent human dendritic sarcoma cell line
Source: Sci Rep. 2020 Dec 4;10:21221. doi: 10.1038/s41598-020-77471-7 (PMC7718904; doi:10.1038/s41598-020-77471-7)
Supplement: Supplementary file 1 — Supplementary Information. [file 41598_2020_77471_MOESM1_ESM.pdf]

U-DCS - characterization of the first permanent human dendritic sarcoma cell line

Kevin Mellert[1], Julian Benckendorff[1], Frank Leithäuser[1], Katarzyna Zimmermann[1], Peter Wiegand[2], Giada Frascaroli[3], Michaela Buck[1], Muriel Malaise[4], Gunther Hartmann[5], Winfried Barchet[5], Daniel Fürst[6], Joannis Mytilineos[6], Regine Mayer-Steinacker[7], Andreas Viardot[7], and Peter Möller[1]*

[1]Institute of Pathology, University Hospital Ulm, Ulm, Germany

[2]Institute for Forensic Medicine, University Hospital Ulm, Ulm, Germany

[3]Institute of Virology, University Hospital Ulm, Ulm, Germany

[4]Department of Pediatric Hematology, Oncology and Stem Cell Transplantation, University of Regensburg, Regensburg, Germany

[5]Institute for Clinical Chemistry and Pharmacology, University of Bonn, Bonn, Germany

[6]Institute of Clinical Transfusion Medicine and Immunogenetics, German Red Cross Blood Transfusion Service, Baden Württemberg-Hessen, Ulm, Germany

[7]Department of Internal Medicine 3, University Hospital Ulm, Ulm, Germany

Supplementary Information

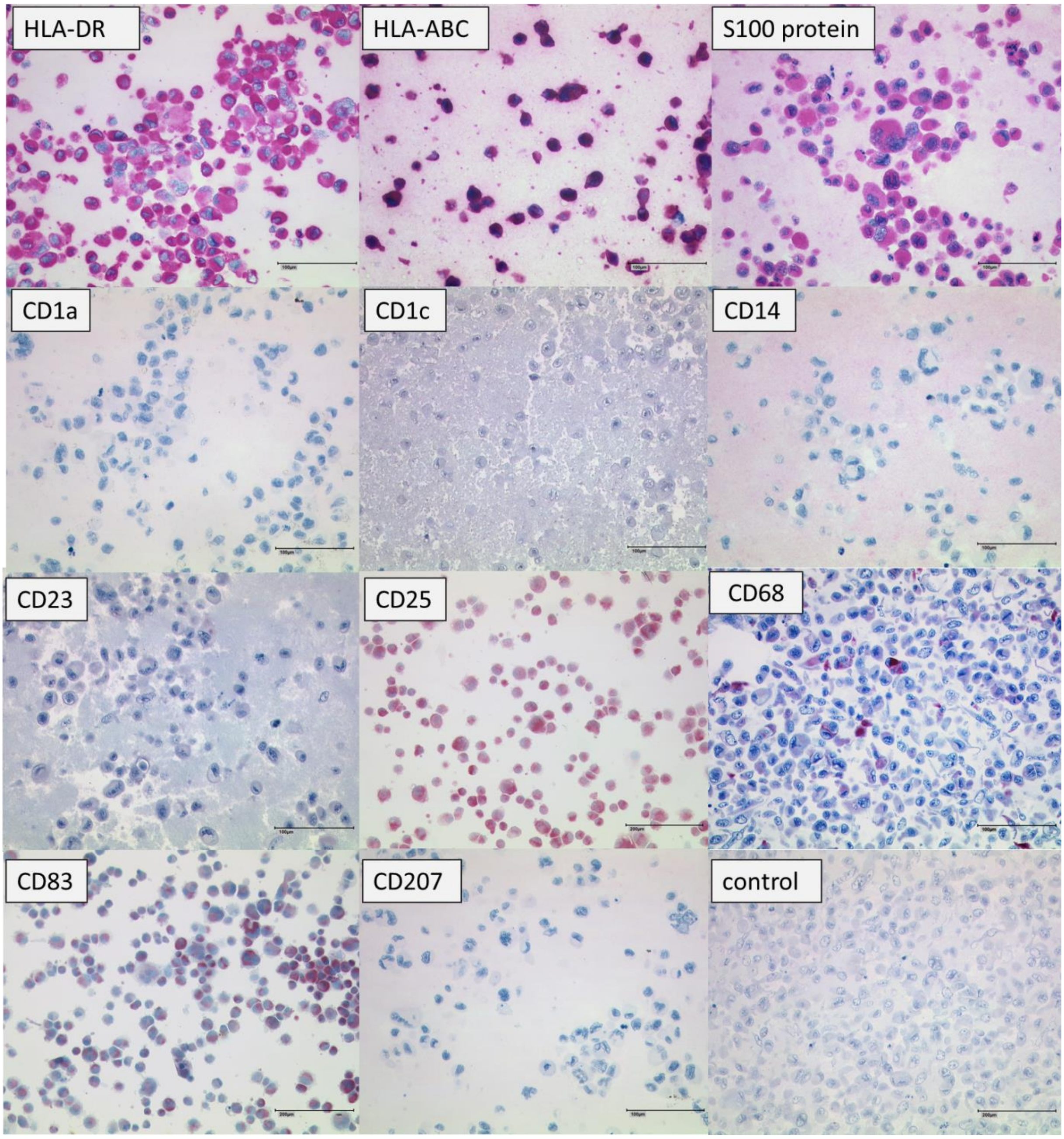

Supplementary Figure 1: Immunocytostaining of U-DCS cytoblock sections. 2µM sections were immunostained with the indicated antibodies. The cells are strongly positive for HLA-DR, HLA-ABC, S100 protein, positive in varying intensities for CD68 and negative for Cd1a. CD1c, CD14, and CD207. The marker line indicates a length of 100µM. "Control" is the result of a negative control without primary antibody.

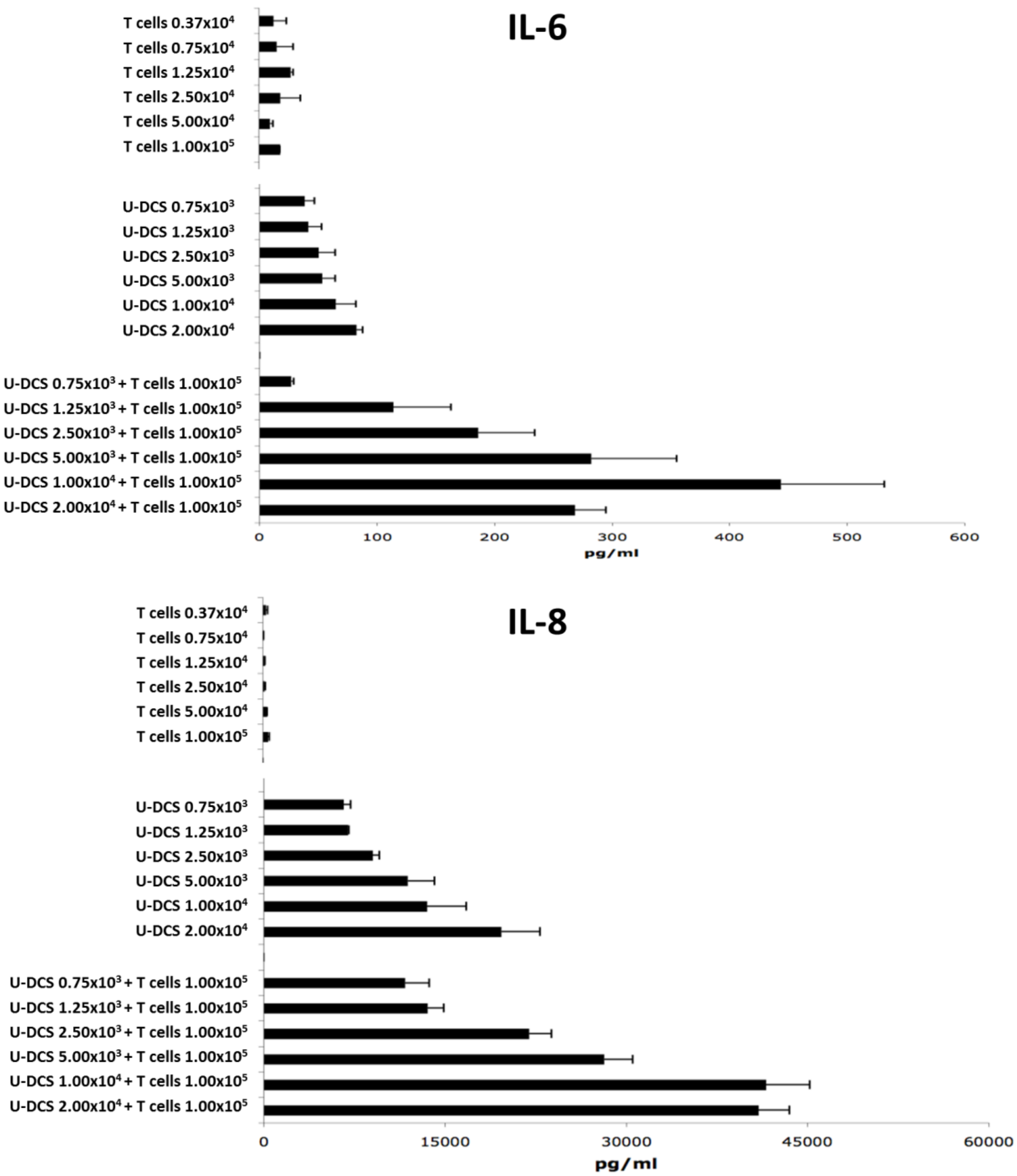

Supplementary Figure 2: Measurement of amounts of Interleukin 6 and 8 in differing numbers of T-cells, U-DCS cells and co cultured cells. Measurements are shown as means with SD (n≥3).

Supplementary Table 1: Detection methods, tested material and primer sequences to detect endogenous viruses.

**Virus**	**Cell line/ patient/ material/test method**		**Primer (5' – 3')**
ß-Actin (used as control)	cell line, PCR	sense	tacatggctggggtgttgaa
		antisense	aagagaggcatcctcaccct
ADV (adeno-associated viruses)	cell line, PCR	sense	actacaayattggctaccagg
		antisense	caaaacataaagaagkgtgggc
		nested sense	aacttccagcccatgagcmg
		nested antisense	ctcaaaagtcatgtcbagcgc
CMV (cytomegalovirus)	cell line, IH		
EBV (Epstein Barr virus)	cell line, PCR, CISH	sense	ctttaaaactctaaaaatcaaaactttag a
		antisense	accagaaatagctgcaggaccactttat ac
		nested sense	aatggcgccattttgt
		nested antisense	tccctagaactgacaatt
HHV-6 (human herpesvirus 6)	cell line, PCR	sense	catcgcatacgtctcccag
		antisense	tctctaacgtgtccgtgcc
		nested sense	cccattggaactgtggtct
		nested antisense	tagagatatgcactcaccg
HPV (human papillomaviruses)	cell line, PCR, IH	My09	cgtcc(ac)a(ag)(ag)gga(at)actga tc
		My11	gc(ac)caggg(at)cataa(ct)aatgg
PVB19 (parvovirus B19)	cell line, PCR	sense	agcatgtggagtgagggggc
		antisense	aaagcatcaggagctatacttcc
		nested sense	gccaactctgtaacttgtac
		nested antisense	aaatatctccatggggttgag
ADV	Patient/feces/Elisa		
Astroviruses	Patient/feces/Elisa		
CMV	Patient/blood/virus isolation - infectiosity		
Hepatitis B virus	Patient/blood/Elisa/anti HBc and HBs Ag		
Hepatitis C virus	Patient/serum/Elisa/ anti HCV		
HHV-6 [51]	Patient/blood/P CR	HHV6-AB-P	aagacctaaattgccgctac
		HHV6-AB-M	gcaagctcatgaacatcgtca
		HHV6-AB-probe	FAM- ttagatggtggtgagctgggatcggt- TAMRA
HIV (human immunodeficiency virus)	Patient/serum/Elisa/p24Ag/antibody		
Noroviruses	Patient/feces/Elisa		
PVB19	Patient/blood/P	Parvo-VP2R	ctgaagtcatgcttgggtatttttc

[52]	CR	Parvo-VP2F	tggcccattttcaaggaagt
		Parvo-probe	FAM-ccggaagttcccgcttacaac-TAMRA
Rotavirus	Patient/feces/Elisa		

Supplementary Table 2: Primer sequences for the end-point measurement of the expression of DDX58 (RIG-1), TLR2, -3, -4, -9 and HPRT.

**Target gene**		**PCR primer sequence (5‘-3‘)**
**DDX58 (RIG-1)**	forward	TTGCTACGTGGTCAACAACA
	reverse	CAAAAGAGCATCCAGCAACA
**TLR2**	forward	TGATGCTGCCATTCTCATTC
	reverse	CGCAGCTCTCAGATTTACCC
**TLR3**	forward	ACCCATACCAACATCCCTGA
	reverse	GCCCTCAAAGTGGATGAGAA
**TLR4**	forward	TGAGCAGTCGTGCTGGTATC
	reverse	CAGGGCTTTTCTGAGTCGTC
**TLR9**	forward	CTACGATGCCTTCGTGGTCT
	reverse	GCCCACAGGTTCTCAAAGAG
**HPRT**	forward	CTTGCTGGTAAAAGGACCC
	reverse	ATTCAAATCCCTGAAGTATTCATTATAGTC
